# Facilitators of the health advocacy role practice of the nurse in Ghana: A qualitative study

**DOI:** 10.1002/hsr2.220

**Published:** 2021-01-10

**Authors:** Luke Laari, Sinegugu Evidence Duma

**Affiliations:** ^1^ Department of Nursing and Midwifery Presbyterian Nursing and Midwifery Training College Bawku Ghana; ^2^ College of Health Sciences University of KwaZulu‐Natal Durban South Africa

**Keywords:** facilitators, health advocacy, nursing and Ghana, role practice

## Abstract

**Background:**

Identifying facilitators of health advocacy role practice of nurses is important in reducing health disparities and inequities in Ghana. The struggle to reducing these disparities and inequities needs a combination of bravery, courage, and professionalism. In many instances, many barriers hinder nurses from practicing their health advocacy role in Ghana. Facilitators that motivate nurses who would perform this health advocacy role have not been identified and adequately described in Ghana.

**Aim:**

To explore and describe the facilitators of the health advocacy role of nurses in Ghana.

**Methods:**

This qualitative study used Strauss and Corbin's grounded theory approach to collect and analyze data from 2018 to 2019 in three regions in Ghana. Semistructured interviews (*n* = 24) and field notes were used to collect data.

**Results:**

Professional influence emerged as a core category among other three facilitators that motivate nurses to perform the health advocacy role. The other three are clientele influence, intrinsic influence, and cultural influence.

**Conclusions:**

Facilitators to the health advocacy role practice of nurses are multidimensional and hidden. In this respect, educating hospital managers on these facilitators should be done through workshops and seminars to enhance the managers' strategies of motivating nurses to advocate for the less privileged and the disadvantaged of the society.

## 1 INTRODUCTION

Nurses are health advocates who safeguard their clients' autonomy, act on behalf of clients and champion social justice in the provision of healthcare.^1^ The dearth of literature to facilitators for health advocacy role practice is aligned to most nurses' inability to speak up for the less privileged and the disadvantaged. The silence of the nurse in the health system has the potential to perpetuate disparities and inequities in an already fractured health system in Africa. These prevailing health disparities and inequities in some African countries including Ghana increase the demand for health advocates. Nurse managers working in these fractured health systems require information to facilitators for the health advocacy role, to motivate nurses in situations that demand the nurses' intervention. As health advocates that show impartiality and courage in speaking up, nurses health advocacy role is required when vulnerable clients are needing the nurses' protection.^2^, ^3^ To conduct themselves in this fashion, nurses need to remain motivated and informed. However, the dearth of literature for factors that facilitate and enhance nurses' diligent practice of their health advocacy role is lacking. This qualitative study explored, identified, and described facilitators nurses in Ghana used in performing their role as health advocates.

## 2 BACKGROUND

Literature on health advocacy role by nurses in underdeveloped and developing countries in Africa is lacking. Facilitators to the health advocacy role practice by nurses are defused in Africa leaving nurse managers with no information on what would have facilitated nurses to advocate. In developed countries, the literature has identified a wide range of contextual conditions and resources that help to make health advocacy possible. This includes education, professional development, and leadership training for the health workforce.^4^, ^5^ Cultural sensitivity, the orientation of the relevant stakeholder groups, communication, action‐oriented mindsets, and dissemination of information have also been identified as equally significant in facilitating health advocacy role practice among health practitioners.^6^, ^7^ Josse‐Eklund et al^8^, ^9^ reported five facilitators that are relevant in influencing health advocacy role practice. These include the nurse's character traits, the nurse's bond with the client, the organizational and cultural facilitators, and the nurse's desire to prioritize disadvantaged and vulnerable clients. The literature further reports empathy, understanding, being sympathetic with and feeling close to the patient, prioritization of patients' health needs, commitment to giving holistic care, and protection of patients' rights as elements that facilitate the health advocacy role practice.^10^ In addition to the intrinsic facilitators, Barlem et al^3^ argue that personal values and individual's professional skills are major sources of support and motivation for the practice of the health advocacy role. This view of personal values is supported by Dadzie et al^11^ who reported that being empathetic, nurturing, ethical, assertive, persistent, and compassionate is found to facilitate health advocacy role practice among nurses. Dadzie et al^11^ further reported that their participants perceived health advocacy as a moral responsibility and identified nurses with excellent communication skills as a significant facilitator in health advocacy.

For health advocacy to be more productive, nurses need to be assertive rather than aggressive in their communication style.^12^ Speaking up as a professional requires an informed and knowledgeable workforce,^13^, ^14^ more than the nurse speaking his or her mind. Speaking up requires knowing and understanding the best practices and scientific rationale.^15^ Speaking up as a nurse is an essential role to provide expert knowledge about clinical practices that result in safe, effective, and high‐quality care.^16^ A study by Black^17^ underscores the need for a shift in organizational culture toward one that encourages clear and open communication by nurses when patient safety may be in jeopardy, indicating the significance of communication in the nurse's role performance. Choi^18^ and Erickson^19^ believe that providing insights into how nurses practice health advocacy in healthcare settings and the facilitators on how they may develop this role further is significant in facilitating the health advocacy role.

Other authors have focused on specific facilitators of advocacy for health equity. Raphael et al^20^ argue that a strong ideological commitment of health professionals to a structural view of the social determinants of health in our society would motivate nurses to speak out. Farrer et al^21^ have explained Raphael et al^20^ argument and identified long‐term facilitators to effective advocacy for health equity, including improving public understanding and awareness of health inequities, the inclusion of advocacy, human rights, and social justice as part of professional training for nurses. Also, authors like Pálsdóttir et al^22^ argue that more critical student contact with disadvantaged communities during their studies would facilitate health advocacy role practice.

The literature from developed countries has highlighted the importance of understanding and appreciating the facilitators that enhance the health advocacy role. However, there are still considerable gaps in knowledge of these facilitators within the African context. This article aims to address this gap and contributes new knowledge that can be used by nurse managers to motivate the nursing workforce to advocate.

## 3 METHODS

### 3.1 Study design

This study used an inductive descriptive qualitative design with grounded theory as a methodology based on Strauss and Corbin (1990). A descriptive design was used because of its suitability in identifying issues with current nursing practice to justify and make a recommendation as proposed by Grove et al.^23^ The design, therefore, was appropriate for the objective of this study, which was to explore and describe the facilitators of the health advocacy role of nurses to make recommendations.

### 3.2 Study area

The study was carried out in Ghana, where three selected regional hospitals were chosen representing three of the 16 regions. This was purposively carried out to include participants from the upper, middle, and southern parts of the country. This was also done to ensure that the findings were accepted by all nurses in the various ethnic groups across the country as the range and scope of nurses' duties are slightly different depending on the location and culture where each of these hospitals is located. The selection was also meant to facilitate constant comparison during data analysis, which is a crucial feature in grounded theory. Regional hospitals were used because they were equally equipped with similar infrastructure and human resources, thus ensuring similarity even though located in different geographical cultural settings. These hospitals selected serve as referral, research, and teaching hospitals where health professionals of various categories are trained.

### 3.3 Sampling

A combination of two sampling techniques was used to recruit participants for this study. These methods were open and theoretical sampling techniques, which are the main sampling techniques in grounded theory.^24^ Open sampling involved the selection of nurses who met the inclusion criteria in the initial stages of data collection. The researchers recruited five nurses in each of the three regional hospitals for open sampling. After open sampling of the initial sample of 15 nurses, transcripts were analyzed to guide theoretical sampling. For open sampling, each hospital, the researchers asked for a list of all nurses who have worked there for more than 5 years, registered with the Nursing and Midwifery Council and were not facing any disciplinary charges from their employer or the Nursing and Midwifery Council. All those who met these criteria were invited for a meeting where the research objectives were explained, and those who consented were recruited. This saw initial recruitment of the 15 nurses for the open sampling. After analyses of the 15 participants nurses and categorizing, some categories appeared less densified, necessitating theoretical sampling.

Theoretical sampling was based on the concepts derived from the evolving categories to assist in comparisons during preliminary data analysis. The main purpose was to determine events that maximized opportunities, and this aided in the discovery of variations among concepts to densify categories in terms of their properties and dimensions.^24^ To densify these categories, three additional participants from each of the three selected regions were considered purposively based on the emerging categories as the theoretical sample. This means that the researchers continuously identified and compared emerging categories of nurse's views on their understanding of facilitators and returned to the field and recruited more participants to extend categories of the emerging categories.^25^, ^26^ Thus, theoretical sampling allowed the researchers to look for data through the views of nurses who knew the facilitators of health advocacy, resulting in nine participants being considered for theoretical sampling.

### 3.4 Sample size determination

The sample size for this study followed the concept of saturation, a major principle of qualitative research.^27^ A total of 24 nurses were recruited in all. First, 15 nurses were recruited for open sampling. After open sampling, to densify the categories, nine nurses were recruited using theoretical sampling, making a total sample size of 24 participants. This was an appropriate sample size for qualitative research to obtain and generate sufficient data, as supported by Charmaz.^28^, ^29^

### 3.5 Data collection process

The interviews were conducted once with each participant by the first author, who is a male nurse educator and holds a PhD in nursing. A one‐on‐one semistructured interview guide was used to collect data, as the interview questions asked were based on the research objective. The semistructured interviews approach was useful as it allowed for in‐depth probing while allowing the interviewer to keep the interview within the limits outlined by the objective of the research. Participants were asked, what in their opinion would make a nurse take actions on behalf of the client/community when cases of health inequities are observed? And followed up with what influences your health advocacy role to a client or a community?

The interviews were audio‐recorded with the help of a trained research assistant who also kept the time for the interviews. The interviews were conducted in English to facilitate the transcription and analysis of data. Data collection lasted for 7 months (August 2018 to February 2019). Field notes were made and kept by the researcher during the interviews and used as part of the data for analysis. Each interview lasted for an average of 120 minutes and was carried out in the ward sisters' offices of the selected hospitals. Participants were not compensated for participating.

### 3.6 Data management and analysis

The recorded interviews were transcribed verbatim within 24 hours after the interviews by the researchers and saved in Word documents. These were read over methodically to carefully review the contents for analysis and refine the interview questions to ensure that the phenomenon under investigation was clear and understandable following the principles of Noiseux et al.^30^ After cleaning the transcripts, data analysis was done immediately using colored pens, paper, and sticky notes, which provided a platform to maintain constant interaction with the data. These were subsequently imported into the QSR international NVivo version 12 computer software to facilitate storage and quick retrieval. The initial data analyzed assisted constant comparative analysis and theoretical sampling. The general guidelines used for data analysis were from the Strauss and Corbin^31^ framework, and Duma.^32^ This recursive line‐by‐line data analysis was carried out following the paradigm model, which, according to Strauss and Corbin^31^ entails three stages, namely, open coding, axial coding, and selective coding. Even though these are mentioned as units on their own for clarity, the researchers perform these synchronously between the open and axial coding in most cases.

### 3.7 Ethical considerations

Before starting data collection, ethical clearance was sought and obtained from the UKZN Ethics Review Committee in South Africa and the GHS Ethics Review Committee in Ghana, with approval numbers HSS/0289/018D and GHS‐ERC 007/05/18, respectively. The study information sheets were given, and the objectives explained to the participants. Participants were assured of their freedom to participate and to leave anytime without prejudice before signing informed consent forms. Anonymity and confidentiality were adhered to during and after data collection. Participation was voluntary.

### 3.8 Scientific rigor and trustworthiness of the study

Trustworthiness in qualitative research is judged through the criteria of credibility, confirmability, dependability, and transferability.^33^ These strategies were applied as discussed below.

The credibility of the findings of the current study was ensured through member checking, where the researchers went back to nine of the participants with the transcripts for their validation and confirmation. Confirmability was ensured as raw data and codes, including field notes, were presented to the coauthor for inputs and to perform the audit trail. In addition to confirmability, the services of an intercoder were used with peer debriefing, as recommended by Morse.^34^ For dependability, data analysis procedures and other complementary data analysis procedures adopted in the study and the actual application of each of the processes in the study are provided to show the accuracy in the implementation of the principles of the grounded theory methodology. An audit trail, which involves outlining the decisions made throughout the research process to provide a rationale for the methodology and interpretative judgment of the researcher,^35^ was also implemented. To maintain transferability, researchers have provided a piece of background information about the participants, the research context, and setting to allow others to make transferability judgments.

## 4 FINDINGS OF THE STUDY

### 4.1 Participants

The study participants were nurses who were then registered with the regulatory body in the country to practice and were working in the selected three regional hospitals. These 24 nurses had a minimum qualification of a diploma in registered general nursing and a maximum of a PhD, but the majority were first‐ and second‐degree holders. The participants' ages were between the ages from 22 to 58 years (mean = 42, SD = 13). Seven participants were male and 17 were female. Years of nursing experience ranging from 5 to 36 years (mean = 24, SD = 11).

The findings emerged with four categories as facilitators from data namely, clientele influence, intrinsic influence, professional influence, and cultural influence (Table 1).

**TABLE 1:** Summary of findings of facilitators to the health advocacy role

Categories	Subcategories
Clientele influence	Clientele readiness Clientele vulnerability
Intrinsic influence of the nurse	Empathy for the client Personality traits of the nurse The passion of the nurse
Professional influence	Knowledge level of the nurse Job satisfaction Professional obligation
Cultural influence	Religious obligation Societal norms requirements

### 4.2 Clientele influence

The nurses expressed that clientele readiness and clientele vulnerability are factors that motivate them to perform the health advocacy role. Client's readiness to be assisted, client openness to ask for help, desire to be supported by the nurse, and their attentiveness to the nurse were expressed as facilitators of health advocacy role practice as demonstrated below:

You might feel that the patient needs someone to stand up for him, you get closer, and he is not ready to open up…The openness of the client, for me, is the first step to advocacy (PN02, 39‐year‐old female).

Sometimes, the actions of the patient can also influence you the staff, to advocate for them…if the client is attentive to what you are saying it is motivating to the nurse to want to address all the patient's concerns (PN06, 31‐year‐old male).

Clientele vulnerability was expressed as a state of being of the client that propels the nurse to speak out for him or her. Participants saw clientele vulnerability as individuals who have no one to speak for them, and a dejected individual in the ward, as demonstrated from participants data below:

You feel pity for the client because he has no one to visit and looks dejected in the ward (PN06, 31‐year‐old male).

I saw the client, and how vulnerable he was, I felt sorry for him and decided to stand up and be his voice (PN17, 40‐year‐old female).

### 4.3 Intrinsic influence

The innate traits of the nurse that facilitates their health advocacy role practice were considered as an intrinsic influence. The nurses reported empathy as the ability to understand and share the situation of the client. This intrinsically influences and drives the nurse to perform the health advocacy role such as being a sister keeper and the need to help “a fellow man” as depicted in the extracts below:

You feel for the patients, and putting yourself in the patients' situation and circumstances, you are in haste to help them because you will be sad for them (PN14, 37–year‐old female).

Sometimes we are just our sister's keepers because they are just weak, you can see that they are suffering, especially, those in their 3rd trimesters of pregnancy are weak, so empathising [with them], you will want to stand up and speak for them (PN09, 42‐year‐old female).

Apart from empathy, personality traits of nurses were also expressed by the participants as facilitators to health advocacy role practice. This intrinsic influence that facilitates the health advocacy role practices of the nurse was reported as inner motivation to advocate, while others talked about it as the intuition of the nurse to speak out. Participants also expressed affability and approachability of the nurse as facilitators.

It is the inner satisfaction I get, internal motivation, it is in me, and because of that, I will always advocate (PN01, 33‐year‐old male).

Yeah, individual characters and affability of the nurse facilitate advocacy, a patient might relate with you very well because of the way you behave towards him or her…being approachable draws clients close and creates the opportunity to speak out for them (PN11, 41‐year‐old male).

Being passionate to help others, having a passion for the work, and recognizing the need to advocate were reported by participants as facilitators to health advocacy, as demonstrated by extracts from participants below:

I think nursing is all about passion…what else can push you than the desire or the passion for helping somebody? …is just the passion (PN07, 43–year‐old female).

I think it's just passion, I might not be wealthy, but I can do something little to touch someone, that's just my philosophy, that's what drives me so it's just passion (PN13, 43‐year‐old female).

### 4.4 Professional influence

The inspiration that a nurse gets to advocate based on his/her professional obligation was expressed. Participants reported a knowledge level of the nurse and that his/her ability to use the skills acquired through experience or education. It encompasses a theoretical and practical understanding of the profession which the nurse has gathered during years practice. Most of these were expressed as knowing what should be done and having adequate knowledge of observation skills. The extracts below indicate this:

So, I think to know that this should be done, and this is what I can do, is a key facilitator to advocate (PN19, 41‐year‐old female).

The knowledge of the condition or situation and then knowing what the person needed at that moment… Also, knowledge and skills of observation, if you can identify that this person needs this sometimes, it helps in speaking for them (PN16, 31‐year‐old female).

Participants reported job satisfaction as a facilitator that professionally influences their health advocacy role practice. The feeling of fulfillment or contentment of the nurse after performing the health advocacy role, being satisfied with accomplishing a role, and receiving thanks from the client, seeing the recovery of the client, and seeing the patient gaining holistic health professionally influence and motivate to advocate. The comments below reflect this from some participants.

The satisfaction that comes after speaking out for an individual with a problem is enough motivation to keep you speaking out for the disadvantaged (PN20, 33‐year‐old male).

To see somebody, recover…and tell you, ‘Thank you, madam’…to see every day somebody is recovering, and his needs are met holistically, that's just the secret motivation, as a nurse, to advocate (PN07, 43‐year‐old female).

Some nurses saw the role as a professional obligation and as such a facilitator to advocate. The moral and legal bounds to the nurse to perform the health advocacy role. The participants perceived professional obligation as facilitators to health advocacy because getting justice for all and maintaining a healthy society is a professional obligation which every nurse is zealous about. The extracts below show this.

It a professional obligation, done based on humanitarian grounds, the nurse‐patient relationship is like families, so whatever we do to see to it that the family is well taken care of is our duty (PN07, 43‐year‐old female).

I believe in justice for all, and I was motivated by my zeal to ensure justice for every client regardless of their background, that is where I get my motivation to advocate (PN03, 36‐year‐old female).

### 4.5 Cultural influence

Some nurses mentioned societal norms and religious beliefs as facilitators to the health advocacy role. They saw the health advocacy role as a religious obligation and societal norm requirements and as such performed most advocacy roles as their culture required. Conceiving it as a religious obligation made them treat clients based on the golden rule of the Bible (do unto others what you wish they would do to you). They did so to fulfill a religious obligation and maintained a clear conscience. The comments below demonstrate this:

For me, I see it as some religious obligation, yeah to speak out for someone to help them get what they couldn't get on their own (PN20, 33‐year‐old male).

If I were in their shoes, what would I like somebody to do for me? Those are the things I considered, and that is why I will always advocate (PN17, 40‐year‐old female).

Other participants reported that what motivates and facilitates their health advocacy role is based on societal norms. In a society, some rules are required by each of us to follow. As such, we are doing our part as members of society. Some participants' comments below reflect this:

We don't live alone; we live with others in society, and rules are governing every society…so sometimes we advocate obeying those rules (PN03, 36‐year‐old female).

The societal norms are the yardstick that facilitates this role (PN19, 41‐year‐old female).

## 5 DISCUSSION

The present study aimed to explore and describe facilitators to the health advocacy role practice of nurses in Ghana. The described facilitators to health advocacy role of the nurse offer the nurse and the nurse manager a substantial insight into areas of health advocacy facilitators. These facilitators were categorized into four main influencers from the perspective of the nurses.

Clientele influence comprising of client readiness and vulnerability were issues nurses considered and rendered the health advocacy role. The nurses interviewed believed that clients' own readiness to be assisted was a necessary influencing factor for health advocacy role performance. Clients' openness to ask for help or calling on the nurse to explain their current situation was emphasized. These findings are consistent with Barlem et al,^3^ where the vulnerability of a client was reported as elements that facilitated the advocacy role practice. Similar findings were also reported by Josse‐Eklund et al,^9^ in which nurses reported that the desire to prioritize the disadvantaged and vulnerable motivated their actions during health advocacy. Our findings suggest nurses in this study either had a challenge initiating conversation with clients or were time constraint and hence sought for openness from clients to facilitator their advocacy role.

Apart from the clientele influence, the nurses believed that inborn attributes, empathy, and passion of the individual nurses were expressed as influencers to the health advocacy role practice. These intrinsic influencers are congruent with previous studies where passion and empathy of individuals were reported as factors that influence the nurse's ability to advocate.^10^, ^11^ These authors reported nurses' ability to understand and share in the feeling of the client, thereby assisting the client in obtaining optimum health, was a motivation. The importance of an inborn desire to care (intrinsic influence) brings lights to explain why nursing is considered a calling,^36^ and only individuals with inner desire to care should be selected to pursue nursing as a carrier. Unfortunately, how to determine the calling or the inner desire during selection remains a challenge.

The professional influence which emerged as a core category of our finding had links with good observation skills, knowing what to do and showing confidence as a professional. It appeared from our findings that knowledge and educational level increase an individual's desire to perform the health advocacy role. This is suggested because participants with a minimum of a first degree reported having played more advocacy roles with little obstacles. This confirms Hanks^13^ reports that lack of education about health advocacy is a barrier and that knowledge of what and how to perform advocacy during training might improve the role practice. Both Rainer^12^ and Beyea^16^ also reported the significance of knowledge, noting that is not about speaking out but knowing what to speak out about is the utmost. In this respect, coaching and mentoring nursing students on how to advocate will facilitate their performance of the role. Job satisfaction was expressed by many strongly that it gives nurses the energy to continue playing this role. Notwithstanding the numerous challenges they are confronted with, the satisfaction nurses get from the performance of this role keep them moving. Seeing clients progress or recover brings joy and job satisfaction. They considered it a motivating force to maintain a healthy society as previously reported by other authors,^8^, ^9^ as were their responsibilities as professionals, confirming Albina^2^ views. Some nurses saw the role as a professional obligation as previously reported.^2^, ^3^, ^13^, ^15^, ^21^ The nurses interviewed believed that there were situations where they felt obliged to perform the health advocacy role based on the principle of saving lives and providing justice to all.

Culture as an integral part of our society was not left out. The nurses believed culture influences who perform the health advocacy role. Religious obligation and societal norms (grouped as cultural influence) were expressed as facilitators to the health advocacy role practice. Those nurses who saw health advocacy role performance as a religious obligation treated their clients based on the golden rule of the Bible. They did so to fulfill a religious obligation and to have and maintain a clear conscience after work. This finding was found as additional information to the factors that influence health advocacy role practice in Ghana.

### 5.1 Limitation

Data collection for this study was done in regional hospitals located in the regional capital; nurses in private hospitals and clinics in the rural areas of the country were not interviewed. Information from these categories would have given more insight into the topic.

## 6 CONCLUSION

Facilitators of the health advocacy role are multifactorial emerging from the professionals' intrinsic traits, clientele, and cultural influences. In all the facilitators, the professional plays the central role as he/she serves as a link to the other facilitators to accelerate health advocacy role performance. There is a need for health facility management to offer continuous professional development to nurses to update their knowledge in health advocacy role performance and to empower them to speak out. Continuous professional education of nurses should be encouraged, as the findings suggest that educational level influences the nurse ability to advocate.

## RECOMMENDATION AND FURTHER RESEARCH

Based on these findings, the authors recommend further studies on religion and the health advocacy role, especially in Ghana.

## FUNDING

The authors received financial support from the College of Health Sciences of the University of KwaZulu‐Natal, scholarship fund for data collection.

## CONFLICT OF INTEREST

The authors declare no conflicts of interest.

## AUTHOR CONTRIBUTIONS

Conceptualization: Luke Laari, Sinegugu Evidence Duma

Data curation: Luke Laari, Sinegugu Evidence Duma

Formal analysis: Luke Laari

Writing ‐ original draft preparation: Luke Laari, Sinegugu Evidence Duma

Writing ‐ review and editing: Sinegugu Evidence Duma

       All authors have read and approved the final version of the manuscript.

       Luke Laari had the full access to all of the data in this study and takes absolute responsibility for the integrity of the data and the accuracy of the data analysis.

## TRANSPARENCY STATEMENT

Luke Laari affirms that the manuscript is an honest, accurate, and transparent account of the study being reported; that no important aspects of the study have been omitted; and that any discrepancies from the study as planned have been explained.

## Data Availability

Data for this study are the property of the University of KwaZulu‐Natal and can be made available upon request from Luke Laari via email laariluke@yahoo.com.
